# The Development Pathway for Biosimilar Biotherapeutics

**DOI:** 10.18502/jovr.v15i3.7444

**Published:** 2020-07-29

**Authors:** Marco Zarbin

**Affiliations:** Institute of Ophthalmology and Visual Science, New Jersey Medical School, Rutgers University, Newark, USA

Many physicians have experience using generic drugs in their practice. Generics tend to be small molecules with a relatively simple structure and are identical to licensed reference products. Generic compounds typically are synthesized using organic medicinal chemistry. Variations in the manufacturing process are unlikely to have a major impact on the final product, an outcome that is verified through analytical characterization of the generic.

A biosimilar biotherapeutic is a protein. Biosimilar products are quite different from generic drugs. A biosimilar has similar quality, safety, and efficacy to a licensed reference product, but it is not necessarily identical to the reference product with regard to these properties.^[1]^ In contrast to generic drugs, biosimilars tend to have complex structures and may differ from the reference product in their primary amino acid sequence and other features such as glycosylation and PEGylation that alter their tertiary structure (i.e., protein folding) as well as their immunogenicity.^[2]^ These differences arise from the manufacturing process, which tends to be much more complex than that of generic drugs. Typically, a biosimilar protein is synthesized by transfecting a target cell with a DNA sequence that encodes the desired product. Often, transfected mammalian cells are required to produce complex proteins, but these cells typically have lower yields than bacterial hosts. The initial product must be purified to remove undesired proteins. As one might expect, the use of different expression systems can be associated with different post-translational protein modifications.

Changes in the manufacturing process are thus critical (and essential to avoid patent infringement on proprietary biomanufacturing processes), as they may alter protein structure and function. Analytical characterization of these compounds is not straightforward and, in any case, is not expected to reveal structure and properties identical to the reference product if different vectors, expression systems, purification steps, and excipients are used in the manufacturing process. Analysis of the immunogenicity of a biosimilar, for example, is a critical aspect of evaluating the therapeutic modality, whereas a generic drug is expected to be identical to the reference product in this regard. The development process and quality control for biosimilars are challenging,^[3,4]^ which helps to explain why the manufacturing costs for biosimilars and generic drugs are quite different, averaging $100–200 million/molecule for the former and $3–5 million/molecule for the latter. Accordingly, the price reduction for biosimilars versus generic drugs is less and might be on the order of 20–30%.

Clinical trials of biosimilars must demonstrate safety and efficacy comparable to the reference product regarding pharmacokinetic, pharmacodynamic, and immunogenic properties. If phase 3 studies are successful and a biosimilar is approved for one indication, it is approved for all other indications for which the reference product is approved, provided there is adequate scientific justification.^[5,6]^ In general, there is an expectation that a patient can switch from a biosimilar to the reference product and vice versa with no lapse in therapeutic efficacy or increased risk. Switching studies demonstrating interchangeability of biosimilars and reference products have not been required for marketing approval by the European Medicine Agency,^[6]^ whereas they are required by the US Food and Drug Administration (US FDA) (https://www.fda.gov/media/124907/download). In order to assist physicians in identifying biosimilars versus the reference product and avoid inadvertent product substitution, the US FDA determined that each biosimilar's name should comprise a core name hyphenated with a four-letter suffix representing the developer.^[7]^

Studies such as the one reported by Lashay and coworkers in this issue of the *Journal of Ophthalmic and Vision Research* constitute an essential step toward adopting use of Stivant, a biosimilar to bevacizumab, for non-approved ophthalmic indications.^[8]^ This work has been executed expertly and provides reassurance that Stivant may well be an appropriate substitute for intravitreal bevacizumab, which is more expensive. The authors qualify their results with great care, but it may be worth emphasizing a few points. First, the rabbit retina is merangiotic and has no fovea. Apart from the inability to identify drug effects on foveal function, these and other features of the rabbit eye may lead to differences in the intraocular and systemic pharmacokinetic profile of the drug associated with intravitreal injection in human patients. Also, although the dose administered was much higher than that anticipated for human subjects, a dose response curve was not undertaken. While we may conclude that a dose of 1.25 mg in a normal size human eye is likely to be safe, we do not know the upper bound of a safe dose based on the data provided. Naturally, these animals had healthy eyes. We do not know whether the safety profile observed in this work will be the same in eyes with damaged retina, retinal pigment epithelium, and/or choroid, as will be encountered in patients with diabetic retinopathy, retinal vein occlusion, and age-related macular degeneration. Of course, these same limitations apply to studies using bevacizumab in rabbits, but biosimilars can differ in subtle ways from the licensed product they mimic. Nonetheless, the data provided by Lashay and coworkers are positive and justify additional studies that will enable Stivant to be deployed for clinical use in patients with retinal vascular diseases. Ultimately, efforts such as these will enable us to provide sight-saving therapy to many more patients through the cost savings realized by the use of biosimilars. I commend the authors for this excellent work and look forward to additional progress in this area. We and our patients will benefit enormously from their efforts.

##  Financial Support and Sponsorship

Nil.

##  Conflicts of Interest

There are no conflicts of interest.
